# Deskilling and labour market barriers among skilled racialized immigrants in British Columbia: a mixed-methods study

**DOI:** 10.1186/s40878-025-00504-5

**Published:** 2026-01-03

**Authors:** Karun Kishor Karki

**Affiliations:** School of Social Work, University of British Columbia, Vancouver, Canada

**Keywords:** Deskilling, Skilled immigrants, Employment integration, Occupational mobility, Intersectionality, Labour market, British Columbia, Canada

## Abstract

This study examines the deskilling experiences of skilled, racialized immigrants who immigrated to Canada through the Federal Skilled Worker (FSW) program and resided in the Lower Mainland of British Columbia. Skilled immigrants, particularly racialized immigrants, continue to face barriers in the Canadian labour market, resulting in significant *deskilling*, which has negative implications for the country’s economy and skilled immigrants. This mixed-methods study drew on a quantitative survey (*n* = 111) and qualitative focus group discussions (*n* = 18) to examine the complex dynamics contributing to deskilling. Multiple linear regression was used to analyze the quantitative data, and thematic analysis was used for the qualitative data. The quantitative results revealed that deskilling was significantly associated with the non-recognition of foreign credentials, upgrading education in Canada, bridging social capital, and having a master’s degree as their academic qualification. The qualitative findings revealed three overarching themes: (a) the illusory nature of meritocracy in employment, (b) deficiencies in social and community resources, and (c) gendered pathways to deskilling. This study provides a comprehensive understanding of the systemic and gendered barriers faced by skilled racialized immigrants in British Columbia’s labour market. It emphasizes the importance of revisiting current immigration policies to better align with labour market realities and address these inequalities.

## Introduction

Over the past two decades, a growing body of scholarly literature has examined economic immigration in Canada (Doyle et al., 2025; Ertorer et al., 2022; Lu & Hou, 2020). A significant policy shift occurred with the enactment of the *Immigration and Refugee Protection Act* (IRPA) in 2001, followed by revisions to the merit-based Point System in 2002. These changes marked a turning point in Canada’s approach to immigration, placing strong emphasis on selecting immigrants based on their human capital, such as education, professional experience, and language skills (Lu & Hou, 2020; Thomas, 2023). As a result, Canada’s immigration system increasingly prioritized highly skilled immigrants with the qualifications needed to address specific labour market demands (Banerjee et al., 2025; Elrick, 2022; Karki, 2020, 2025). This policy orientation is reflected in the rising number of economic migrants, which increased from 159,289 in 2017 to 252,971 in 2021, indicating the country’s growing reliance on highly educated and skilled immigrants to meet the labour market needs (Immigration, Refugees and Citizenship Canada, 2022).

Although skilled immigrants are admitted through Canada’s merit-based immigration system, which emphasizes human capital attributes such as education and professional experience, their labour market trajectories are frequently constrained by systemic barriers that inhibit the full utilization of their professional skills and expertise. This results in the phenomenon of “deskilling,” which should not be understood as an isolated outcome but rather as a structural process embedded within institutional mechanisms that systematically devalue foreign-earned credentials and professional experiences. Empirical evidence indicates that within four years of arrival, few immigrants achieve recognition of their educational credentials or professional experience (Houle & Yssaad, 2010; Cox, 2023). Professions regulated by licensing bodies, such as engineering, medicine, and law, accentuate these difficulties through gatekeeping mechanisms that privilege Canadian credentials (Bauder, 2003; Cox, 2023). Immigrants trained abroad, particularly in fields such as engineering, are significantly more likely to experience *occupational mismatch*, and this disparity intensifies when intersected with race and gender (Konnikov, 2023). Additionally, systemic biases, such as “foreign-sounding names”, result in lower callback rates for highly skilled applicants (Diversity Institute, 2020). These findings reveal that deskilling is not a residual effect of volunteer credentialism but a targeted manifestation of institutional privilege.

Deskilling is shaped by the intersection of race, gender, class, and migration status, with racialized women facing distinct barriers to recognition and career advancement (Karki, 2025; Liu, 2019). Despite high educational attainment, racialized immigrant women are disproportionately employed in low-wage, precarious, and part-time jobs (Momani et al., 2021). The *Skills Next* report highlights their significant wage gaps and underrepresentation in leadership roles (Public Policy Forum, 2020). Beyond economic exclusion, deskilling among racialized immigrant women is linked to adverse health outcomes, increased caregiving burdens, and social isolation, illustrating its broader socioeconomic impacts (Premji & Shakya, 2017). Intersectionality thus reveals how overlapping oppressions produce layered forms of marginalization. Deskilling must also be situated within broader neoliberal and racialized labour market regimes. Bauder (2006) employs labour market segmentation theory to argue that migrant labour is intentionally devalued by regulatory and cultural forces, thereby preserving opportunities for native-born professionals. This aligns with literature on precarious immigrant employment, which demonstrates that neoliberal immigration policies commodify skilled migrants while denying equitable access to stable and appropriate work (Karki et al., 2018; Liu, 2019).

The immigration literature has focused on the occupational change experienced by skilled immigrants in low-skilled jobs (often with low earnings) that are not aligned with their educational and professional backgrounds (Creese & Wiebe, 2012; Lightman et al., 2022; Reitz, 2005; Slade, 2012). This phenomenon has been conceptualized in various ways in the literature, including downward occupational mobility (Salami & Nelson, 2014), under-employment (De Jong & Madamba, 2001), brain abuse (Bauder, 2003), skill erosion (Galgóczi et al., 2016), over-qualification (Chen et al., 2010), job mismatch (Banerjee et al., 2018), and skill underutilization (Anwar, 2014; Reitz, 2005). These studies consistently show that many skilled immigrants, particularly those from Asia, the Caribbean, the Middle East, and Africa, immigrate to countries in the Global North with the expectation of securing employment commensurate with their educational qualifications and work experience. However, they often encounter structural forms of exclusion, such as non-recognition of foreign credentials, discrimination, and limited access to professional networks, which prevent them from achieving their goals. Unlike non-migrant populations and white immigrants, their skills are often perceived as inferior, prompting them to seek and accept employment that underutilizes their human resources and competencies (Creese & Wiebe, 2012; Pung & Goh, 2017; Sert, 2016).

The phenomenon of racialization has been examined by Guo (2015) and Bauder (2003) within the context of multicultural societies such as Canada, highlighting how immigrants’ skills are stratified based on race. This stratification diminishes and devalues qualifications and professional backgrounds acquired abroad. In this study, I use the term “deskilling” to describe situations in which skilled, racialized immigrants are employed in occupations unrelated to their qualifications and experience or in roles that require their qualifications but are at a significantly lower level. This often necessitates reskilling, which does not always lead to positive outcomes, such as upward occupational mobility in the labour market. Scholarly literature documents why immigrants are often compelled to work in occupations that do not match their educational qualifications and work experience (Bauder, 2003; Creese & Wiebe, 2012). Several factors influence the labour market integration of skilled racialized immigrants, both facilitating and hindering their transition, including structural barriers, language proficiency, and gender (Creese & Wiebe, 2012; Karki & Neupane, 2021; Sweetman, 2004). For instance, Creese and Wiebe (2012) demonstrated that the devaluation of immigrants’ skills is partly due to the non-transferability of professional credentials across national borders, particularly from countries in the Global South. Some studies have attributed this issue to regulatory systems designed to protect the interests of domestic professionals (Guo, 2009; Nwoke & Leung, 2021).

However, Sweetman (2004) argued that the perceived lower quality of education in migrants’ home countries also contributes to the reassessment and subsequent devaluation of their academic credentials. Language barriers further limit the access of skilled, racialized immigrants to high-skilled employment and hinder their ability to build professional networks, thereby constraining opportunities for upward occupational mobility (Liu & Guo, 2021; Teo, 2007; Lai et al., 2017). Additionally, the literature highlights the complexity added by gender roles. For example, social reproductive responsibilities often push skilled immigrant women into low-skilled jobs that do not reflect their qualifications or prior work experience (Elrick & Lightman, 2016). Lightman et al. (2022) argue that these challenges are shaped by an intersectional process, in which race, gender, and immigration status collectively influence how skilled immigrants navigate the labour market in host countries.

Despite the growing body of research on skilled immigrants, many studies have continued to treat this population as a homogeneous group (Banerjee et al., 2024; Guo, 2015; Liu & Guo, 2021; Man & Chou, 2020; Nardon et al., 2022; Zhang, 2022). However, I argue that skilled immigrants, particularly those who migrated to Canada under the Federal Skilled Workers (FSW) program, constitute a distinct group. These individuals hold what can be considered a “global driver’s license,” which, in theory, should enable them to market their skills in any suitable labour market. They are often regarded as “people without borders” and are seen as possessing significant human capital. Therefore, the tendency to categorize all skilled immigrants under a single label should be reconsidered. Greater attention must be paid to their heterogeneity, particularly with respect to immigration category and race, to fully understand and explain their experiences of employment integration. Moreover, existing studies on immigrants’ employment integration are primarily focused on the national level and within Canada’s three largest metropolitan cities – Montreal, Toronto, and Vancouver (MTV) – while neglecting mid-sized urban and rural centers (Drolet & Teixeira, 2022; Zarifa et al., 2019). This study focuses on the Lower Mainland of British Columbia, a region of approximately 3.05 million people, 54.5% of whom identify as belonging to a visible minority group (Metro Vancouver, n.d.). The research site encompasses the Metro Vancouver regional districts – 21 municipalities including the City of Vancouver and Tsawwassen First Nation – as well as six municipalities in the Fraser Valley, such as Langley, Abbotsford, and Chilliwack (Fraser Valley Regional District, n.d.). Mid-sized urban centers in the Lower Mainland have experienced a growing influx of immigrants, driven in part by government policies designed to promote a more balanced geographic distribution of newcomers (Blower, 2020; Preston et al., 2023).

Using a mixed-methods research design, this study examines and explores the phenomenon of deskilling among skilled racialized immigrants in the Lower Mainland of British Columbia. Specifically, the study aims to (1) examine the extent and nature of deskilling among skilled racialized immigrants and (2) describe their lived experiences of deskilling. This paper is organized into six sections, including an introduction. The second section outlines the theoretical framework for guiding this study. The third section details the research methods, and the fourth presents the findings. The fifth section discusses the results, while the sixth section offers policy recommendations and concludes with suggestions for improving employment integration among skilled racialized immigrants.

## Theoretical framework

This study draws on the theoretical insights of intersectionality (Crenshaw, 1989, 1991; Collins, 1990, 2000) and critical race theory (Delgado & Stefancic, 2023), both of which foreground the structural embeddedness of inequality and emphasize the epistemic significance of lived experience in illuminating power asymmetries. The rationale for integrating intersectionality and CRT into this study is twofold. First, this integrated framework provides a theoretically rigorous and politically grounded lens for examining the structural violence embedded in the Canadian labour market. Second, it enables a methodological praxis that resists reductionism and essentialism by centring the embodied, situated knowledge of marginalized groups. This framework interrogates how race, class, gender, and other social categories intersect to produce and sustain forms of exclusion that are institutionally entrenched yet often rendered invisible by dominant discourses of liberal multiculturalism and meritocracy. As Anthias (2012) contends, intersectionality is not simply a mapping of categories but a critical methodology for understanding the dynamic interplay between social locations, institutional arrangements, and identity formations.

Intersectionality, initially conceptualized by Crenshaw (1989) within Black feminist legal scholarship, emerged as a critique of single-axis frameworks in anti-discrimination law and feminist theory. Crenshaw’s intervention exposed the epistemic and political erasure of Black women’s experiences, arguing that the simultaneity of racism and sexism creates distinctive forms of marginalization irreducible to either dimension alone. Intersectionality thus demands a departure from additive models of oppression, offering instead a structural and relational framework to understand how interlocking systems of domination shape social positions and institutional outcomes. Patricia Hill Collins (1990, 2000) extends this critique through her articulation of the “matrix of domination,” which conceptualizes power as multidimensional and relational, operating across structural, disciplinary, hegemonic, and interpersonal domains. Her emphasis on standpoint epistemology centers the knowledge of those situated at the intersections of subordination, contending that these perspectives are not only valid but necessary to apprehend the operations of power. This epistemological orientation is integral to this study, which privileges the voices of skilled, racialized immigrants whose labour market experiences are systematically obscured by technocratic discourses of “skills mismatch.” Rather than viewing deskilling as an individual failure or bureaucratic oversight, this research situates it as a racialized mechanism of labour market stratification, where institutional processes, such as credential devaluation, racialized constructions of competence, and linguistic gatekeeping, undermine immigrant integration (Creese & Wiebe, 2012; Girard & Bauder, 2007). Intersectionality, in this context, functions as both a diagnostic and analytical tool to unpack how these processes are differentially experienced based on the convergence of race, class, gender, and immigration status.

In tandem, critical race theory (CRT) deepens the structural critique by asserting that racism is not aberrational but a normalized and systemic feature of social institutions (Delgado & Stefancic, 2023). CRT challenges liberal narratives of objectivity and neutrality, emphasizing that race is socially constructed yet materially consequential. Scholars such as Aylward (1999) and Crenshaw et al. (1995) argue that racism constitutes the ideological scaffolding of Euro-American-Canadian societies, shaping policy, legal norms, and institutional practices in ways that perpetuate racial hierarchies. There is, as they contend, no neutral ground from which to observe racism; it is embedded within the very structures that govern everyday life (Aylward, 1999; Crenshaw et al., 1995). Within this framework, intersectionality is not limited to identifying multiple identities but is employed as a structural analytic to interrogate how macro-level forces – such as immigration policy, labour market regulations, and neoliberalism – converge to shape micro-level experiences of exclusion. For example, racialized immigrants’ devaluation in the Canadian labour market is not incidental but indicative of how whiteness and Canadian-born experience are constructed as normative, while “foreign” knowledge is delegitimized (Block & Galabuzi, 2011).

This theoretical framework also informs the study’s methodological design. During data collection, purposive sampling targeted participants whose identities reflect multiple axes of subordination, including race, immigrant status, and occupational field. This aligns with McCall’s (2005) intra-categorical approach, which investigates complexity within marginalized groups that are often treated as analytically homogeneous. The use of qualitative, semi-structured interviews is guided by the intersectional imperative to foreground narrative nuance, reflexivity, and multiplicity. In the analytical phase, intersectionality provides a critical vocabulary to examine how categories such as race, class, and migration status are not discrete but co-constitutive. Participants’ narratives reveal how racialized perceptions of “foreign” professionalism intersect with neoliberal discourses of soft skills and cultural fit, reinforcing systemic deskilling despite formal qualifications. These findings highlight how institutions function as racialized gatekeepers, regulating access to employment and professional recognition.

## Methodological approach

### Study setting

The study was conducted in the Lower Mainland, in the southwestern part of British Columbia, Canada. This region is one of the most ethnically diverse areas of the country. With a population of approximately 3 million, the Lower Mainland accounts for approximately 61% of British Columbia’s total population (Trade and Invest BC, 2024). It is recognized as one of North America’s most dynamic and diverse economies, offering abundant economic opportunities. As of 2022, the region’s labour force numbered 1.8 million, with a participation rate of 67.2% and an unemployment rate of 5.1% (TransMountain, 2022). The Lower Mainland also includes seven medium-sized urban centers, including Surrey, Coquitlam, Langley, Maple Ridge, Abbotsford, Mission, and Chilliwack. These centers are experiencing a significant influx of immigrants, largely driven by government policies promoting the geographic dispersion of newcomers to ensure a more balanced distribution across smaller urban and rural areas (Drolet & Teixeira, 2022). Notably, approximately 24% of recent immigrants to British Columbia now reside in these seven centers alone (NewToBC, 2024). Additionally, these communities face demographic challenges, including an aging population and the outmigration of youth to larger cities. These factors highlight the importance of attracting and retaining immigrants in smaller urban settings. The study’s focus on the Lower Mainland, including these medium-sized centers, also provides valuable evidence-based insights, as immigrants in these locations encounter distinct challenges, such as limited transportation infrastructure and retention difficulties, which are often overlooked in research focused on major metropolitan areas (NewToBC, 2018, 2024).

### Study design

A concurrent parallel mixed-methods design was employed to facilitate a comprehensive examination of the deskilling experiences of skilled racialized immigrants in the Canadian labour market. This design enables the simultaneous collection and analysis of quantitative and qualitative data, thereby allowing for a multidimensional understanding of the phenomenon. The quantitative component provides measurable insights into employment outcomes, income disparities, and credential recognition among skilled, racialized immigrants. Concurrently, focus group discussions enabled a deeper exploration of the participants’ lived experiences, illuminating the emotional, social, and structural dimensions of deskilling. The integration of these data sources through triangulation enhanced the validity of the findings and enabled a richer interpretation of the complex interplay between systemic labour market barriers and individual adaptation processes. This methodological approach supported the development of a more nuanced and holistic understanding of deskilling, with implications for informing targeted policy interventions and support mechanisms for skilled racialized immigrants in the Canadian context.

### Data collection

#### Quantitative phase

In the quantitative phase of the study, data were collected through an online survey administered through SurveyMonkey. The questionnaire was developed in English. A graduate student research assistant assisted in designing and programming the SurveyMonkey survey. The survey was pilot tested before it was administered. It took approximately 20 min to complete the survey. Recruitment efforts included disseminating a survey flyer featuring a QR code and a direct survey link through immigrant settlement, service agencies, and social media platforms. These agencies were asked to distribute the materials to potential participants within their networks. Using a combination of criteria and convenience sampling, eligible participants were defined as skilled, racialized immigrants who: (a) immigrated to Canada under the Federal Skilled Workers (FSW) program; (b) were the principal applicants under the FSW program; (c) landed directly from abroad through a Canadian Mission; (d) resided in the Lower Mainland of British Columbia; and (e) self-identified as members of a visible minority group, as defined by the Government of Canada. Individuals who arrived in Canada through alternative immigration streams (e.g., student visas, business visas, temporary work permits) and later obtained permanent residency under the FSW program by applying from within Canada were excluded. This exclusion criterion was applied to ensure that the sample reflected individuals who had arrived from abroad and whose initial adaptation experiences were distinct from those already living in Canada prior to gaining permanent residency. Recruitment was supported by community organizations, including settlement agencies, religious institutions, and ethnic community groups within the study area. These organizations helped disseminate survey materials within their respective networks. This community-based approach helped foster trust and encouraged participation among racialized immigrant populations.

#### Qualitative phase

Four focus group discussions were conducted in the qualitative phase with 18 participants, comprising 10 women and eight men. These discussions aimed to explore in greater depth the lived experiences of skilled, racialized immigrants, particularly in relation to the themes identified in the quantitative data. The participants in the focus groups were recruited through an online survey. Respondents who expressed interest in participating in follow-up discussions were considered for inclusion based on the specified criteria and convenience sampling. This approach ensured that participants met the study’s eligibility requirements while also taking into account logistical considerations, such as availability, location, and willingness to participate in group discussions. Particular attention was paid to ensuring gender balance and diversity within the sample, recognizing that the intersectionality of social identities significantly shapes immigrant experiences, including those related to deskilling and integration. The goal was to capture a wide spectrum of perspectives that would reflect the complexity and heterogeneity of the broader immigrant population. An interview guide was developed based on core research questions and preliminary quantitative findings. This guide served as a flexible framework, ensuring consistency across focus group discussions, while allowing participants to express their experiences on their own terms. Each focus group lasted for approximately 90–120 min. Two sessions were conducted in person, while the remaining two were held virtually via Zoom, aligning with participant preferences and public health guidelines. All sessions were audio-recorded with the participants’ informed consent and transcribed verbatim to preserve the accuracy and integrity of the data. These transcripts constituted the primary data source for subsequent coding and thematic analysis.

### Measurement

#### Outcome variable

In this study, the outcome variable was ‘*deskilling*,’ which is defined as a contextual situation in which skilled, racialized immigrants are employed in occupations that do not correspond to their qualifications and experience, or in roles for which they are qualified but at a level significantly below their credentials. These circumstances often necessitate reskilling/upgrading skills and qualifications, which do not always result in positive labour market outcomes, such as upward occupational mobility. Deskilling was assessed using a six-item seven-point Likert scale. For example, one of the items was, "*I am working in a job not related to acquired skills". *Participants responded to statements on the following scale: 1 = strongly disagree, 2 = disagree, 3 = somewhat disagree, 4 = neither agree nor disagree, 5 = somewhat agree, 6 = agree, and 7 = strongly agree. Composite scores ranged from a minimum of six to a maximum of 42, with higher scores indicating greater levels of perceived deskilling. The scale demonstrated excellent reliability, with a Cronbach’s alpha coefficient of 0.984, indicating strong internal consistency.

#### Predictor variables

Predictor variables were grouped into three dimensions: demographic, socioeconomic, and contextual. The demographic variables included sex, marital status, and age. Socioeconomic variables consisted of educational attainment outside of Canada, length of stay in British Columbia, and whether participants had upgraded their education within Canada. The contextual variables included bonding social capital, bridging social capital, gender discrimination, and non-recognition of foreign credentials. Each of these variables was measured using a custom-developed Likert scale. First, Bridging Social Capital was measured using a five-item scale assessing participants’ sense of closeness to diverse groups, including (a) people who speak different languages, (b) people from different racial or ethnic backgrounds, (c) people who follow different cultural practices, (d) neighbourhood community associations, and (e) immigrant settlement agencies. Responses were provided on a seven-point scale ranging from 1 (very distant) to 7 (very close). Composite scores range from 5 to 35, with higher scores indicating greater levels of bridging social capital. Second, Bonding Social Capital was measured using a four-item scale assessing closeness to culturally similar groups, including (a) people of the same nationality, (b) people who speak the same language, (c) people from the same racial or ethnic group, and (d) people who follow the same cultural practices. The composite scores range from 4 to 28, with higher scores reflecting stronger bonding social capital.

Third, Gender discrimination was measured using a four-item scale examining perceptions of gender-based inequalities experienced by racialized women in the workplace. The items included: (a) racialized women receive fewer opportunities than their male counterparts, (b) racialized women are less likely to be promoted, (c) racialized women are assigned to lower positions due to their gender, and (d) job opportunities for racialized women are restricted because of their gender. The composite scores range from 4 to 28, with higher scores indicating lower perceived gender discrimination. Fourth, the non-recognition of foreign credentials was measured using a four-item scale assessing the recognition of international qualifications and experience: (a) My non-Canadian academic qualifications are recognized at my workplace, (b) My non-Canadian professional experience is recognized at my workplace, (c) My professional skills acquired outside Canada are recognized at my workplace, and (d) My non-Canadian credentials are recognized by credential-assessing bodies in Canada. The composite scores ranged from 4 to 28, with higher scores indicating a greater likelihood of non-recognition of foreign credentials. Cronbach’s alphas for non-recognition, bonding social capital, bridging social capital, and gender discrimination were 0.989, 0.827, 0.771, and 0.818, respectively.

## Analytical framework

Descriptive statistics were used to summarize the characteristics of the study sample for the quantitative data analysis. These analyses included the calculation of proportions, means, and standard deviations (SD). Multiple linear regression analyses were conducted to examine the associations between the predictor variables and the outcome variable, *deskilling*. Multicollinearity among predictor variables was assessed using the Variance Inflation Factor (VIF). All VIF values were below 2.6, indicating no significant multicollinearity issues. The goodness-of-fit of the regression model was evaluated using the Adjusted R², standard error of the estimate, Durbin-Watson statistic, and Cook’s distance. The level of statistical significance was set at *P* < .05. The Statistical Package for the Social Sciences (SPSS) software (IBM SPSS Statistics for Windows, version 27) was used for both descriptive and inferential statistical analyses.

Thematic analysis (Braun & Clarke, 2006, 2022) was employed for qualitative data analysis. This approach facilitated the identification of recurring patterns and themes across the datasets. Data immersion began with transcription, reading/re-reading transcripts, and journaling to capture initial impressions. The next phase involved generating initial codes and developing potential themes by collating relevant coded data. These themes were then reviewed and refined to ensure that they accurately represented the data, both in relation to individual extracts and the dataset as a whole. The final themes were defined and named to convey their underlying meanings. NVivo software (version 15, 2024) was used to support the organization and analysis of the qualitative data. To ensure the trustworthiness of the analysis process, the development of codes and themes, along with their interpretations, was thoroughly reviewed. In doing so, initial codes were generated inductively through line-by-line engagement with the data, ensuring they are grounded in participants’ accounts. These codes were iteratively compared and organized into categories, which were then refined into higher-order themes reflecting semantic and conceptual patterns. Reflexive practices, including analytic memos and iterative review of coding, strengthened the credibility, dependability, and confirmability of the findings, in line with Lincoln and Guba’s (1985) trustworthiness criteria. Finally, quantitative and qualitative findings were integrated in the discussion section, where points of convergence and divergence themes were identified. This integrative approach offers a more comprehensive understanding of the research problem by leveraging the strengths of both data types.

### Ethical considerations

Ethical approval for the study was obtained from the Research Ethics Board of the University of British Columbia (Protocol No: 101494). Written informed consent was obtained from all participants prior to their participation in the study.

## Results

This section presents the results of the survey and the focus group discussions. The quantitative results from the survey are presented first, followed by the qualitative findings derived from a thematic analysis of the focus group discussions.

### Quantitative results

A total of 120 responses were received in this study. However, due to incomplete responses, five were excluded, resulting in a final sample size of 111 participants included in the analysis. Table 1 presents the socio-demographic characteristics of the participants. The mean age of the participants was 40 years (*SD* = 5.0), and the majority were male (55.9%). Approximately 82% of the respondents were married, and 8.1% held a doctoral degree. Most participants were employed full-time (62.7%). In terms of ethnicity, 29.7% (*n* = 33) of the participants identified as South Asian. Among them, 38.9% reported having an educational background in health sciences and technologies. Regarding language proficiency, 52.3% of the participants reported having very good knowledge of English, whereas 99.1% (*n* = 110) indicated limited knowledge of French. The average duration of residence in British Columbia was eight years (*SD* = 3). Participants reported a mean annual income of CAD 61,542 (*SD* = 36,456), indicating a wide range of economic circumstances in the sample.

**Table 1:** Socio-demographic characteristics of the study sample

Socio-demographic characteristics	*n* = 111
Age in years (mean, *SD*)	40 (5)
Gender	
Male	62 (55.9%)
Female	49 (44.1%)
Marital status	
Unmarried	20 (18%)
Married	91 (82%)
Highest level of education	
Professional degree (MD, LLB, or equivalent)	11 (9.9%)
Master’s degree	91 (82%)
Doctoral degree	9 (8.1%)
Employment status	
Full-time	69 (62.7%)
Part-time	41 (37.3%)
Ethnic origin	
South Asian	33 (29.7%)
East Asian	27 (24.3%)
African	6 (5.4%)
Southeast Asian	12 (10.8%)
Middle East	13 (11.7%)
Latin, Central and South American	17 (15.3%)
Caribbean	3 (2.7%)
Knowledge of English	
Good	37 (33.3%)
Very Good	58 (52.3%)
Excellent	16 (14.4%)
Knowledge of French	
Very limited	110 (99.1%)
Very Good	1 (0.9%)
Duration of stay in British Columbia in years (mean, *SD*)	8 (3)
Annual income in CA$	61,542 (36456)

#### Factors related to the deskilling of skilled racialized immigrants

A multiple linear regression analysis was conducted to identify the variables that predict deskilling among skilled, racialized immigrants in British Columbia’s labour market. The predictor variables included gender, marital status, age, educational attainment outside Canada, length of stay in British Columbia, upgrading of education in Canada, bonding social capital, bridging social capital, experiences of gender discrimination, and non-recognition of foreign credentials. The results of the regression analysis are presented in Table 2.

**Table 2:** Regression results predicting deskilling (*n* = 111)

Predictor	B	SE	β	t	VIF
(Constant)	16.06	7.77	–	2.07*	
Gender (Ref. Male)	-2.01	1.27	-0.08	-1.58	1.802
Marital Status (Ref. Unmarried)	-0.62	1.29	-0.02	-0.48	1.123
Age	0.15	0.15	0.06	1.03	2.030
Educational outside Canada (Ref: Doctoral degree)					
Professional degree (MD, LLB, or equiv.)	4.32	2.43	0.11	1.78	2.402
Master’s degree	4.06	1.94	0.13	2.10*	2.517
Length of stay in British Columbia	-0.15	0.19	-0.04	-0.77	1.353
Upgrade Edu. in Canada (Ref. No)	-11.30	1.33	-0.44	-8.50***	1.759
Bonding social capital	-0.12	0.15	-0.03	-0.82	1.173
Bridging social capital	-0.51	0.11	-0.24	-4.57***	1.898
Gender discrimination	0.19	0.11	0.07	1.71	1.239
Non-recognition of foreign credentials	0.67	0.09	0.35	7.10***	1.663

*p ≤ .05; ***p ≤ .05

As presented in Table 2, the analysis indicated that upgrading one’s education in Canada (*β* = -0.435, *p* < .001) and higher levels of bridging social capital (*β* = -0.243, *p* < .001) were significantly associated with lower levels of deskilling. In contrast, greater non-recognition of foreign credentials (β = 0.354, *p* < .001) and holding a master’s degree obtained outside Canada (*β* = 0.128, *p* = .039) were significantly associated with higher levels of deskilling among skilled, racialized immigrants in British Columbia’s labour market. The overall regression model was statistically significant, *F*(11, 99) = 51.95, *p* < .001, explaining 85.2% of the variance in deskilling (*R*² = 0.852, Adjusted *R*² = 0.836). The standard error of the estimate was 4.94, and the Durbin-Watson statistic was 1.82, indicating no significant autocorrelation issues. These findings indicate the critical role of social capital, educational upgrading, and credential recognition in shaping labour market outcomes for racialized immigrant professionals.

### Qualitative results

Among the 18 skilled racialized immigrants interviewed (10 females and eight males), the length of stay in British Columbia ranged from 11 to 19 years. Fourteen participants were from Asia, three from Africa, and one from South America. In terms of pre-migration education, 15 participants held master’s degrees, two had bachelor’s degrees, and one possessed a college diploma. Prior to migration, participants had accrued four to eight years of professional experience in fields such as higher education (as professors), psychology, nursing, and optometry. However, the post-migration employment outcomes revealed significant downward mobility. While one participant remained unemployed, the remaining 17 were employed in low-skilled occupations, including factory work, security services, food and beverage services, front desk administration, and cleaning. A thematic analysis of the interview data revealed three major themes concerning deskilling among skilled racialized immigrants: (1) the mirage of meritocracy in employment integration, (2) failures of social and community resources, and (3) gendered pathways to deskilling.

### The mirage of meritocracy in employment integration

This theme critically examines the disjuncture between the idealized notion of meritocratic opportunity in Canada and the lived realities of skilled racialized immigrants facing structural barriers. The sub-themes discussed in this section include the non-recognition of foreign credentials, the pursuit of reskilling, and the perceived value of volunteering.

#### Non-recognition of foreign credential

Fifteen participants identified the non-recognition of foreign credentials as a pivotal factor leading to their deskilling in the labour market in British Columbia. This group consisted of eight females and seven males; 12 were from Asia, and three were from Africa. All 15 participants had been residing in Canada between 11 and 19 years. Before migration, they had professional work experience ranging from four to eight years, and their educational qualifications included 12 with master’s degrees, two with bachelor’s degrees, and one with a college diploma. Participant 1 shared how struggles with credential recognition led to feelings of invalidation: *“These things haunt me: credential recognition. Who are you to recognize my credentials? My very question is this. What is Canada that it has to recognize my credentials?”* This quote highlights the tension surrounding credential validation processes, illustrating the broader systemic barriers that question legitimacy and perpetuate unequal power dynamics within the labour market. Another participant voiced concern over the paradox of immigration policies that attract skilled migrants only to devalue their qualifications upon arrival:

*My degree was there*,* professionally*,* I was treated very well back home*,* and the degree was a top-most degree in the country*,* and I had a very nice job. But when I came here*,* they think your degree is not Canadian*,* you should have a Canadian degree*,* and Canadian work experience. If you need everything Canadian*,* then why do they need Asian*,* African or other immigrants?*

This perspective challenges the foundational logic of skilled immigrant selection policies when juxtaposed with devaluative practices. The participants’ narratives highlight the perceived disconnect between Canada’s immigration policies and labour market practices, which often leaves skilled immigrants unable to access employment that matches their qualifications.

#### Reskilling without reward

Ten participants (four men and six women) sought to overcome deskilling by upgrading their education in Canada, often seeing it as a pathway to re-entering the professional workforce. For many participants, reskilling was a strategic move to “get a foot in the door” within the Canadian labour market. As one participant shared, “*So upgrading education was a good idea to put my foot in the door. So*,* when I came here*,* I went to university and did an MSc in Vision Science.*”

However, the participants consistently reported that reskilling did not facilitate access to employment commensurate with their *overall* educational qualifications and pre-migration professional experience. One of the participants described pursuing a Canadian degree specifically to overcome employment barriers and only to encounter further disappointment:

*I went to MSW with the hope that this degree would help me find a job because you could get some kind of Canadian experience while doing your placement. And this could be the pathway to my career. But unfortunately*,* it did not work.*

Another participant highlighted the persistent mismatch, even after obtaining Canadian qualifications: “*And after working there for about two years*,* I quit the job and went to college and did a Chartered Professional Accountant (CPA) course. But that degree could not even land me a job in my profession.*” These experiences suggest that reskilling, while sometimes necessary, often fails to adequately counter the complex barriers faced by racialized immigrants, representing a significant investment without anticipated professional return. In other words, these experiences suggest that while reskilling may provide temporary hope, it does not guarantee equitable labour market integration for racialized skilled immigrants.

#### Volunteering dogma and the elusive Canadian experience

Five participants (two men and three women) engaged in volunteer work in an effort to gain “Canadian experience” and improve their labour market prospects. Four were from Asia, and one was from Africa. At the time of the interview, one participant was a healthcare assistant, one was a store manager, two were factory workers, and one was a security guard. While volunteering was often recommended by employment services, these participants described it as largely ineffective at securing skilled employment. One participant described an extensive effort that yielded minimal results.

*People say social networking*,* civic participation*,* volunteering*,* and something like that help you get a job. I have been volunteering*,* did a placement*,* have good relationships with friends from diverse backgrounds*,* and am continuously going to different employment services and settlement organizations.*

One of the participants challenged the efficacy of volunteering as a pathway to employment, highlighting broader experiences of discrimination.

*Some people recommended that I volunteer as a pathway to a job*,* and I did that*,* too. But I know it is discrimination*,* I have experienced so many different forms of discrimination*,* you know. When I came here*,* I did a diploma in psychology*,* I did volunteer for one year*,* I did a co-op.*

Similarly, another participant expressed a deep sense of disillusionment: “*I did CPA here*,* got a license*,* did co-op*,* did some volunteering work too*,* but still not getting a job.*” These accounts illustrate the limits of volunteering as a labour market strategy. While positioned as a mechanism to gain Canadian experience, it often fails to dismantle structural barriers or lead to meaningful employment for skilled racialized immigrants.

### Failures of social and community resources

This theme examines how both formal and informal social networks, although intended to support newcomers, often fail to help individuals find meaningful employment. In many instances, these support systems failed to promote successful integration into the labour market and unintentionally contributed to deskilling. As a result, many participants were unable to apply their existing qualifications or professional experience, leading to underemployment or career setbacks in their new environment. This theme includes two sub-themes: funnelling into low-skilled jobs and bonding social network steering towards deskilling.

In the focus group discussions, most participants (*n* = 13) reported accessing social and community resources upon arrival in Canada, particularly through settlement agencies and social networks. Of these, eight were men and five were women. The majority (*n* = 9) were from Asia, three from Africa, and one from South America. Eleven held a Master’s degree, one had a college diploma, and another held a bachelor’s degree before migration. At the time of the interviews, their employment included food servers (*n* = 4), factory workers (*n* = 5), security guards (*n* = 1), truck drivers (*n* = 1), unemployed individuals (*n* = 1) and receptionists (*n* = 1). Ten participants (seven men and three women) stated that settlement agencies provided inadequate support specifically tailored to the needs of skilled racialized immigrants. They believed the lack of meaningful assistance significantly contributed to their deskilling and hindered their integration into the Canadian labour market. They stated that effective engagement from settlement agencies had the potential to facilitate labour market integration that aligned with newcomers’ existing skills and professional backgrounds, yet their limited support often hindered this outcome. One participant recounted their experience with a settlement agency when seeking employment assistance.

*We also went to settlement services agencies. They tried to help us make our resume. However*,* one thing you know is that the employment counsellor saw my resume and said*,* ‘you are a highly educated person; It is difficult to find a job in your profession.’ So*,* you have to remove the BSc Nursing degree from your resume; otherwise*,* you will be overqualified*,* and the employer will not hire you.*

Another participant expressed disappointment with the limited and at times inappropriate support offered by these agencies.

*I went to a settlement agency and asked if they could help me find a job in dental hygiene. She started asking about training for resume writing. Seriously? Resume writing? I worked on my resume for six months*,* and now again? I need a job. Not a resume writing training. I was really disappointed.*

These narratives indicate a critical gap in the settlement process, where generic, one-size-fits-all services fail to meet the needs of skilled immigrants, who require targeted support to navigate specific professional pathways. As a result, many are left without the guidance or resources necessary to re-enter their fields, leading to underemployment and long-term professional displacement.

#### Funnelling into low-skilled jobs

Seven participants (five men and two women) described that settlement agencies seemed to be primarily oriented toward low-skilled immigrants, inadvertently funnelling skilled racialized individuals into precarious and low-skilled jobs. One of the participants described a jarring transition from professional aspirations to low-skilled recommendations.

*I went to a settlement agency for the first time*,* and I spoke with a counsellor and said I was a lawyer*,* I have a master’s degree in law*,* and more than 5 years of law practice. How can I get a job in my profession? He suggested that I think of grocery stores because I am in need of financial support. He said the jobs in the Law profession are difficult to find here.*

Another participant highlighted the emotional toll of being asked to downplay their qualification. He illustrated how agencies sometimes actively contributed to deskilling by advising participants to conceal their professional background. This practice of concealing qualifications represents direct institutional pressure towards deskilling. He said,

*I went to a settlement agency*,* entered all my information in their system*,* and the employment counsellor asked me to make another appointment after a week. I went there and she recommended that I apply to Tim Horton’s. She totally destroyed my resume. She forced me to make a fake resume by hiding my qualifications and professional experiences. She said employers don’t hire me because I don’t have a Canadian education and Canadian experience. That really bothered me; I was so stressed.*

The lived narratives of participants reveal a poignant paradox experienced by many skilled immigrants: institutions intended to facilitate their integration can often reinforce marginalization and the devaluation of their professional identities.

#### Bonding social networks steering towards deskilling

This theme captures the paradoxical role of close-knit ethnic community ties in the employment trajectories of skilled, racialized immigrants. While these bonding social networks offer essential support and initial job access, participants’ narratives reveal that they often guide newcomers toward low-skilled employment, contributing to a cycle of professional deskilling. These dynamics underscore the limited presence of bridging social capital necessary to connect immigrants with opportunities that align with their qualifications and expertise. Six participants (four men and two women) identified bonding social networks as inadvertently contributing to deskilling. One participant stated the following:

*I consulted with my friends from my community when I came to Canada about how I could enter the job market. They referred me to a private employment agency*,* and the agency helped me find a job in a factory.*

Similarly, another participant described how social connections ultimately facilitated access to low-skilled work: *“Later on*,* with the help of my friends and relatives*,* I got a job in a factory.”* These narratives illustrate how bonding social capital, while providing crucial initial support and community, often channels skilled immigrants into occupational niches characterized by deskilling. While essential for social survival and immediate financial support, it often limits access to broader employment/professional networks and reinforces cycles of under-employment.

#### Gendered pathways to deskilling

This theme explores how gender roles within the home and broader social contexts influence the labour market experiences of skilled, racialized immigrant women. Participants’ narratives revealed that traditional caregiving expectations and cultural norms limiting women’s mobility and networking constrained their access to professional opportunities. These intersecting dynamics of gender, race, and immigration status contribute to distinct pathways for deskilling racialized skilled women. This theme encompasses two sub-themes: the gendered division of labour at home and at work.

Three female participants explicitly discussed the gender implications for their deskilling (two from Asia and one from Africa). All held master’s degrees before migration. Two pursued further education in Canada (one college diploma and one bachelor’s degree). Since arriving in Canada, two had pursued further education, one completing a college diploma and the other earning a bachelor’s degree. In their countries of origin, they hold professional roles as public health workers, senior accountants, and child psychologists. At the time of the interviews, however, they were employed in cleaning, warehouse operations, and food services. The gendered effect manifested primarily through divisions of labour, both at home and in the workplace.

#### Gendered division of labour at home

Two participants indicated that traditional gender norms and the lack of extended family support disproportionately placed the burden of caregiving and domestic responsibilities on women, requiring them to navigate these demands alongside the complexities of the labour market. These responsibilities limited their ability to fully engage in the labour market or pursue career-aligned opportunities. One participant shared her experience negotiating household roles and childcare duties.

*When I was applying for a job in a warehouse*,* my husband was not happy. He insisted that I not work because he was studying full-time*,* and I should be at home with our daughter. However*,* I told him I would manage babysitting*,* so he didn’t have to worry about our daughter. I got a job*,* and it was from 2:00 pm to 10:00 pm. I used to drop off my daughter at her school*,* and the lady used to pick her up and do babysitting until I got home at 10:30 pm.*

This example illustrates the additional logistical and emotional labour required for women to engage in paid work, often constraining their ability to pursue career-relevant opportunities or further Canadian credentialing. This account further reveals that the extra layer of negotiation and coordination women often undertake to balance work and family responsibilities, burdens that may compound the risk of deskilling.

### Gendered division of labour at work

In addition to domestic expectations, cultural norms around gender roles influenced women’s ability to build social capital in the Canadian labour market. One participant explained how cultural expectations shaped her networking practices, which are often seen as essential for professional advancement:

*And their emphasis was to build networking*,* go out*,* and meet people. In my culture*,* it is considered unusual for a woman to be so outgoing. Women still cannot go out as freely as men in public. It was challenging for me to go out and meet people and network here.*

This highlights a key barrier in which strategies for professional integration, such as networking, are culturally restricted or practically challenging for women from certain backgrounds, thus limiting their access to bridging social capital and skilled employment. The participant’s quote illustrates how gendered cultural norms intersect with race and immigrant status to restrict access to informal networks essential for navigating the Canadian labour market. These experiences reveal significant gendered dimensions of deskilling that merit further investigation, especially regarding the interplay between cultural norms, domestic responsibilities, and networking constraints.

## Discussion

This study employed a mixed-methods research design to examine the phenomenon of deskilling among skilled racialized immigrants in the Lower Mainland of British Columbia, Canada. The quantitative results revealed that deskilling was significantly associated with factors such as the non-recognition of foreign credentials, the necessity of upgrading education in Canada, bridging social capital, and holding a master’s degree as an educational qualification. Conversely, the qualitative findings illuminated the more nuanced and experiential dimensions of deskilling. Participants described their disillusionment with the supposed meritocracy of the Canadian labour market, the inadequacy of social and community support mechanisms, and the gendered dimensions of their deskilling trajectories. These findings are consistent with existing literature that identifies credential non-recognition, reskilling, limited access to community resources, and gendered labour divisions as key contributors to the deskilling of skilled racialized immigrants (Creese & Wiebe, 2012; Ghosh, 2020; Karki, 2016; Man & Chou, 2020).

Qualitative data revealed that the non-recognition of foreign credentials played a pivotal role in deskilling skilled racialized immigrants. Participants shared experiences of emotional distress and professional invalidation stemming from the devaluation of their educational and professional background. These narratives align with the quantitative results, where non-recognition of foreign credentials was significantly associated with higher levels of deskilling among skilled racialized immigrants. However, the qualitative insights went further, illustrating the profound psychological toll of being forced to abandon one’s professional identity. This divergence between quantitative and qualitative findings indicates the emotional dimension of deskilling, characterized by frustration, loss of status, and diminished self-worth, which has been echoed in previous research (Guo, 2015; Hari, 2013; Karki, 2020; Zhang, 2022). The lack of recognition of foreign credentials often compels immigrants to accept low-wage and manual employment, thereby exacerbating socioeconomic marginalization (Hari, 2013; Zhang, 2022). As Somerville and Walsworth (2010) note, this contradiction is especially concerning, given that many immigrants are admitted to Canada under a points-based system that emphasizes education and work experience. Despite this, newcomers frequently face systemic barriers to credential recognition, including opaque registration procedures, a lack of clear guidance, a requirement for Canadian experience, and difficulties in passing certification exams (Gyan et al., 2024; Man & Chou, 2020; Shan, 2013). The psychological and social implications of this forced career transformation are significant. According to Guo (2013), the transition from professional to manual labour diminishes immigrants’ social standing and has detrimental effects on mental health and well-being. Consequently, the non-recognition of foreign credentials is not merely a bureaucratic challenge but a structural barrier to full integration, contributing to long-term socioeconomic and psychological marginalization (Karki et al., 2023; Mullings et al., 2020). This study’s findings underscore the implications of non-recognition of foreign credentials for the employment integration and well-being of skilled racialized immigrants in Canada.

The quantitative results further revealed that upgrading education in Canada is associated with significantly lower levels of deskilling. However, qualitative findings complicate this interpretation. Many participants reported that while they engaged in additional education or training in the hope of improving their employment prospects, these efforts rarely resulted in jobs commensurate with their qualifications or previous professional experience. Skilled, racialized immigrants also volunteered to gain Canadian experience and improve their employment prospects, commensurate with their credentials. Nevertheless, volunteering did not translate into employment as employers did not consider it a Canadian experience. This discrepancy suggests that while statistical models may indicate that upgrading reduces deskilling, in practice, such efforts do not guarantee the professional reintegration of skilled, racialized immigrants into employment commensurate with their qualifications and work experience. Existing research supports this complexity, noting that, although many skilled racialized immigrants invest in retraining, they often remain excluded from professional roles (Ghosh, 2020; Hari, 2013; Karki & Moasun, 2023; Liu & Guo, 2021; Sakamoto, 2007).

Liu and Guo (2021) highlighted that settlement counsellors frequently encourage reskilling as a strategy for employment. However, this encouragement often fails to account for the fact that reskilling may necessitate a complete career change, leading many immigrants to move away from their pre-migration fields. While some immigrants secure jobs aligned with their newly acquired Canadian skills (Hari, 2013; Salaff & Greve, 2004), others continue to struggle, leading to unfulfilled career aspirations and the pursuit of alternative, often lower-status employment paths (Maitra, 2015). The findings suggest that, while upgrading and reskilling may reduce deskilling, they often substitute immigrants’ professional identities with alternative career paths. These findings also indicate that deskilling/reskilling often comes at the cost of abandoning an individual’s original professional identity and aspirations.

The quantitative results revealed that bridging social capital was significantly associated with lower levels of deskilling among skilled racialized immigrants. However, qualitative data challenged this finding. The participants in focus group discussions reported that settlement agencies, conceptualized as sources of bridging capital, often failed to facilitate effective labour market integration. Instead of supporting immigrants in leveraging their skills, these agencies sometimes reinforced deskilling by directing individuals towards survival jobs, advising them to conceal their qualifications from resumes, or offering generic guidance that failed to align their professional experiences with the Canadian labour market. This discrepancy indicates that, while bridging capital may be a statistically significant predictor of a lower level of deskilling, its actual effectiveness, as found in qualitative findings, depends significantly on the quality and context of support provided to immigrants and newcomers.

This study revealed that not all forms of bridging capital are equally effective or empowering. Institutional actors, such as settlement agencies, play a crucial role in either enabling or constraining skilled racialized immigrants’ access to meaningful employment in the labour market. The divergence can be reconciled when the quality of bridging capital is considered, as the quantitative measures of the association and the qualitative findings reveal the limitations. Both findings align with those of studies that applaud and critique the services of settlement agencies. (Liu & Guo, 2021) indicated that counsellors offered career guidance that helped immigrants discover their professional identity, which was aligned with their values and personality, and led to personal purpose and career satisfaction. This suggests that bridging capital is an avenue for empowering skilled racialized immigrants. Nevertheless, studies have also found that settlement services funnelled people into low-skilled employment, as they hardly helped with profession-specific skills (Karki, 2025; Sakamoto et al., 2009; Rice & Quan, 2023; Sakamoto, 2007). This reinforces the need for settlement agencies to examine quality, as they perpetuate deskilling as a default pathway without challenging the credential recognition regime. These findings necessitate a critical evaluation of settlement services, particularly in terms of their roles in supporting immigration integration and settlement.

The qualitative findings of this study revealed distinct gendered pathways to deskilling among skilled racialized immigrants, particularly among women. The gendered division of labour within households disproportionately places the burden of domestic responsibilities on women, thereby constraining their participation in the formal labour market. Skilled racialized women often find themselves balancing the complexities of employment integration with unpaid caregiving roles, a dual burden that significantly hinders their professional advancement. The narratives of skilled racialized women indicated that cultural norms and expectations regarding gender roles shaped their deskilling experiences. These findings align with prior research that highlights the gendered dimensions of deskilling among skilled racialized immigrant women (Ghosh, 2020; Man & Chou, 2020; Sakamoto et al., 2009; Teo, 2007), and extend the literature by demonstrating how gendered barriers affect access to professional networks, an area less examined in the Canadian context (Ibarra, 1993; McDonald, 2011). This suggests that women frequently internalize patriarchal power dynamics, which coerce them into assuming primary caregiving responsibilities, often at the expense of their professional development. For instance, Liu and Guo (2021) argue that women often submit themselves to patriarchal power relations, which coerce them into becoming family caretakers. The challenge was even more pronounced for women who were the heads of households. Balancing the demands of childcare, household management, and paid employment has created a situation in which maintaining a professional trajectory has become increasingly complex (Man & Chou, 2020).

The gendered division of labour within a household has significant implications for career advancement and marital relationships, often leading to tension and conflict. The study also found that migration can disrupt previously equitable marital relationships, complicating existing research on working-class women’s migration experiences (Hondagneu-Sotelo, 2000). Skilled racialized women reported tensions arising from shifts in domestic labour and career trajectories, highlighting the intersection of gender, migration, and professional identity. Moreover, Sakamoto et al. (2009) found that women who transitioned to full-time caregiving roles frequently experienced a decline in previously egalitarian marital dynamics, which often gave way to strained and conflict-ridden relationships. This suggests that shifts in domestic roles can disrupt equitable family structures, especially in the context of migration. These insights underscore the need for policy interventions that promote shared domestic responsibilities and provide targeted support, such as affordable childcare and flexible work arrangements, to enable equitable labour market integration for immigrant women. Ghosh (2020) further emphasizes the impact of the “motherhood penalty” and systemic employment barriers, which collectively derail the careers of skilled racialized women.

Gendered dynamics within households and labour markets continue to shape the deskilling experiences of skilled racialized immigrants, particularly women, as migration often disrupts previously equitable marital arrangements and constrains participation in professional networks. Recognition and validation of international qualifications are critical for equitable employment; however, formal credential recognition alone is insufficient (Karki & Moasun, 2024). Access to mentorship, professional networks, and structured pathways for women balancing caregiving responsibilities, alongside high-quality bridging capital, is essential to mitigate deskilling. These intersecting structural, social, and psychological factors underscore the need for integration policies that address credential recognition, gendered barriers, and social capital in a holistic manner. In terms of policy implications, while Canadian reforms have aimed to address credential non-recognition (Banerjee et al., 2025; Karki, 2025), this study suggests that these measures may be insufficient if structural barriers related to gendered network access, household responsibilities, and psychological impacts of deskilling are not addressed. Policies should complement credential recognition with programs that facilitate professional networking, mentorship, and pathways for women balancing caregiving responsibilities. Additionally, interventions addressing the emotional and relational consequences of deskilling, such as disruptions to marital relationships and professional identity, could enhance labour market integration and well-being.

The quantitative findings from this study also revealed that possessing a master’s degree earned outside Canada was significantly associated with higher levels of deskilling among skilled racialized immigrants. These findings highlight the persistent challenges highly educated immigrants face in securing employment that aligns with their academic qualifications and prior professional experience. Although these immigrants possess high levels of human capital, they frequently face systemic barriers to employment, including the non-recognition of foreign credentials, devaluation of international work experience, and a labour market that prioritizes Canadian experience over global expertise. These findings are consistent with previous literature documenting the downward occupational mobility of skilled racialized immigrants – a phenomenon frequently described as “brain waste” (Malik et al., 2017; Lai et al., 2017; Maitra, 2015). Maitra (2015), for example, found that employers in Canada often fail to recognize foreign degrees as equivalent to domestic qualifications, instead demanding additional Canadian certifications or credentials. The findings have implications for the narratives of meritocracy as espoused in the point-based immigration system adopted by Canada to select the best candidates for integration into the labour market. The disjuncture between the selection criteria for immigration and the realities of labour market integration suggests that systemic inequities continue to shape the employment outcomes of skilled racialized immigrants.

## Conclusion

This study employed a mixed-methods approach to investigate the deskilling experiences of skilled, racialized immigrants in the Lower Mainland of British Columbia. The quantitative findings revealed that upgrading education in Canada and higher levels of bridging social capital were associated with lower levels of deskilling. Conversely, the non-recognition of foreign credentials and completion of a master’s degree outside Canada were significantly associated with higher levels of deskilling among skilled, racialized immigrants in Lower Mainland BC. The qualitative findings provided deeper insights, revealing that the illusion of meritocracy in employment integration, the inadequacies of social and community resources, and the gendered division of labour all contributed to the deskilling experiences of skilled racialized immigrants in the labour market. These findings have important implications for both policy and practice. First, there is an urgent need for a comprehensive review and reform of Canada’s credential recognition process. Despite skilled racialized immigrants being admitted under the federal point system, the labour market often fails to recognize their qualifications and experience. Transparent and streamlined credential recognition pathways are necessary to eliminate barriers such as complex procedures, high costs, and a lack of information.

Second, employment support programs should incorporate mental health services to address the emotional and psychological impacts of deskilling, such as the loss of professional identity and reduced self-worth. To promote professional continuity, targeted programs should be implemented to facilitate entry into occupations aligned with the immigrants’ original fields of expertise. This approach would shift away from the current emphasis on requalification, retraining, or career redirection, which is often promoted by settlement agencies. It is also essential that the roles of settlement agencies be re-evaluated to provide more personalized and profession-specific career guidance that empowers skilled racialized immigrants. Labour and immigration policies must also be responsive to caregiving responsibilities that disproportionately affect racialized immigrant women. Employment integration programs should offer flexible work arrangements and targeted interventions, such as mentorship, professional bridging programs, and pathways that accommodate extended periods spent outside the workforce owing to caregiving obligations. Finally, greater investment is needed in mental health and social support services to address the long-term psychological consequences of deskilling, including the emotional toll of losing one’s professional identity and disruption of family dynamics following migration. Addressing these complex and intersecting issues is essential to support the successful integration of skilled, racialized immigrants into the Canadian workforce and society at large.

### Strengths and limitations

This study was conducted in the Lower Mainland of British Columbia, a racially diverse region of over three million residents, more than half of whom identify as members of visible minority groups (Metro Vancouver, n.d.; Fraser Valley Regional District, n.d.). A key strength of the study lies in its mixed-methods design. The quantitative analyses identified associations between credential recognition, educational upgrading, bridging capital, and deskilling, while the qualitative strand offered depth by illuminating the lived experiences underlying these patterns, including barriers to professional integration and the gendered dimensions of labour market participation. The integration of these findings provided a more comprehensive understanding of how structural barriers are both statistically evident and subjectively experienced.

At the same time, the study has limitations. Sampling was non-random, and therefore statistical significance testing was not intended to support claims of generalizability; instead, it was used to identify systematic patterns within the data. The findings should thus be interpreted as exploratory and indicative. This approach aligns with prior research that employs statistical tests with non-probability samples to highlight associations warranting further investigation (Bryman, 2016; Etikan et al., 2016).

## Data Availability

No datasets were generated or analysed during the current study.
